# MedCSS: a causal self-supervised approach for hierarchical feature consistency in 3D medical imaging

**DOI:** 10.3389/fnins.2026.1739716

**Published:** 2026-02-16

**Authors:** Jiang Han, Fei Wang, Xingchen Shen, Feng Cao

**Affiliations:** 1 Institute of Geriatrics, Beijing Key Laboratory of Aging and Geriatrics, National Clinical Research Center for Geriatrics Diseases, Second Medical Center of Chinese PLA General Hospital, Beijing, China; 2 Institute of Software, Chinese Academy of Sciences, Beijing, China; 3 University of Chinese Academy of Sciences, Beijing, China

**Keywords:** causal self-supervision, deep learning, feature consistency, medical image analysis, representation learning

## Abstract

Medical image analysis plays a crucial role in linking perceptual mechanisms with clinical diagnosis, yet conventional deep learning models often rely on statistical correlations rather than modeling the underlying generative structure, leading to limited robustness in small-sample and cross-domain scenarios. To address this issue, we propose a hierarchical feature consistency framework named “MedCSS” that integrates causal self-supervised learning. Built upon a 3D ResNet backbone, the method aligns intermediate and high-level features through distributional consistency while introducing a coding rate–based causal regularization to suppress non-causal redundancy. Experiments on the MedMNIST3D benchmark demonstrate enhanced feature stability, boundary sensitivity, and generalization across diverse medical structures. Visualization analyses further reveal improved morphological coherence and causal interpretability. This study highlights the potential of causal self-supervision for structurally robust and semantically consistent representation learning in three-dimensional medical imaging.

## 1 Introduction

Medical image analysis represents a crucial interdisciplinary field at the intersection of biomedical science and artificial intelligence research. Its core objectives extend beyond assisting clinical disease detection and diagnosis to encompass understanding how biological systems encode, transmit, and integrate perceptual information across multiple scales and modalities (Litjens et al., 2017; Anaya-Isaza et al., 2021). The morphological and dynamic changes in imaging signals reveal tissue structures and functional states, providing an objective foundation for exploring the neural mechanisms underlying perception, attention, and cognitive processing (Greenspan et al., 2016; Tajbakhsh et al., 2020). The widespread application of artificial intelligence technologies, particularly deep learning, enables models to automatically learn underlying patterns from large-scale medical images. This has driven the convergent development of intelligent diagnostic systems and perception modeling.

The introduction of deep learning has shifted medical image analysis from manual feature extraction to end-to-end representation learning (LeCun et al., 2015; Schmidhuber, 2015). Models based on convolutional neural networks (CNNs) have demonstrated outstanding performance in tasks such as organ segmentation, lesion detection, and image reconstruction (Ronneberger et al., 2015; Çiçek et al., 2016; Kamnitsas et al., 2017). However, these approaches also exhibit inherent limitations: First, they heavily rely on large-scale manually annotated data, which is costly to produce and prone to observer bias (Esteva et al., 2021); Second, models primarily establish representations through correlation learning, lacking characterization of the data generation process and structural causal mechanisms; finally, significant distribution differences across modalities and devices substantially impact model generalization performance (Huang and Chung, 2022). In medical video-based dynamic lesion monitoring tasks, existing models struggle to handle image degradation caused by adverse conditions such as noise, rain, and snow, failing to consistently deliver accurate diagnostic information (Liu J. et al., 2025). In scenarios involving dense lesion prediction—such as brain tumors and polyps—traditional hierarchical networks suffer from scale information loss and feature misalignment, directly reducing lesion boundary recognition accuracy and impacting clinical intervention decisions (Liu X. et al., 2025). Therefore, how to obtain representations with causal consistency and structural interpretability under limited annotation conditions represents a core scientific challenge currently facing medical artificial intelligence.

To alleviate reliance on labeled data, self-supervised learning has gradually emerged as a key method for medical image representation learning (Caron et al., 2020; Chen et al., 2020; Grill et al., 2020). Its fundamental approach involves designing proxy tasks—such as contrastive learning, image reconstruction, and rotation prediction—to extract high-level abstract features from unlabeled samples, thereby enhancing feature transferability and data utilization efficiency (Grill et al., 2020; Azizi et al., 2021). Against this research backdrop, the MedMNIST series of datasets (Yang et al., 2023) provides a standardized evaluation platform covering multimodal 2D and 3D tasks, offering a unified benchmark for comparing the performance of different self-supervised and semi-supervised models. However, traditional self-supervised models often capture only statistical commonalities without modeling the underlying generative causal relationships behind observations.

In recent years, the concept of causal learning has been introduced into self-supervised frameworks, giving rise to a new direction known as Causal Self-Supervised Learning (Causal SSL). This approach introduces causal consistency constraints, enabling models to not only learn feature correlations but also focus on generative dependencies and intervention invariance among different variables (Schölkopf et al., 2021). Counterfactual reasoning can effectively mitigate biases, such as the Counterfactual Bidirectional Co-Attention Transformer framework reducing multimodal integration bias through counterfactual scenarios (Ji et al., 2025; Jones et al., 2024)' three-step framework also provides methods for bias identification and fair model development. The Undoing Memorization Mechanism (UMM) framework proposed by Qiang et al. (2025). reveals the memorization bias in self-supervised models and achieves causal stabilization of representations through hierarchical feature distribution alignment and coding rate constraints. Building upon this, the SEMI-CAVA model further embeds causal inference mechanisms into variational semi-supervised learning, enabling the identification of latent causal factors through latent variable intervention (Saha et al., 2025). These studies offer new perspectives on understanding structural representations in models, demonstrating the significant potential of causal constraints to enhance model generalization, interpretability, and cross-domain stability.

At the model architecture level, Residual Networks (ResNet) (He et al., 2016) have become a common backbone for learning representations in 3D medical images due to their efficient gradient propagation and feature reuse capabilities (Chen et al., 2021; Lee et al., 2025). ResNet variants of varying depths can capture multi-level features ranging from local textures to global semantics, providing a solid structural foundation for achieving cross-layer causal consistency learning. Building upon this, this paper proposes a hierarchical feature consistency learning framework based on a causal self-supervised mechanism. Using 3D ResNet as the backbone, it achieves cross-layer causal consistency through feature distribution alignment and coding rate regularization. Its effectiveness is validated on the MedMNIST3D dataset for nodule, vessel, and neural synapse classification tasks. This research not only demonstrates the feasibility of causal self-supervised learning in medical image analysis but also offers a novel computational perspective for understanding causal structure learning in artificial perception systems.The main contributions of this paper include:

- Proposing a 3D medical image classification framework that integrates causal self-supervision with hierarchical feature constraints, enabling the learning of causally consistent representations from unlabeled data;
- Systematically validating its robustness and cross-domain generalization performance across multi-modal structural tasks using the MedMNIST3D dataset family;
- Revealing the role of causal self-supervision in enhancing structural understanding and information compression from a perceptual cognition perspective, providing theoretical support for modeling biological perception mechanisms in artificial intelligence.

## 2 Methods

### 2.1 Overview

**Figure 1:**
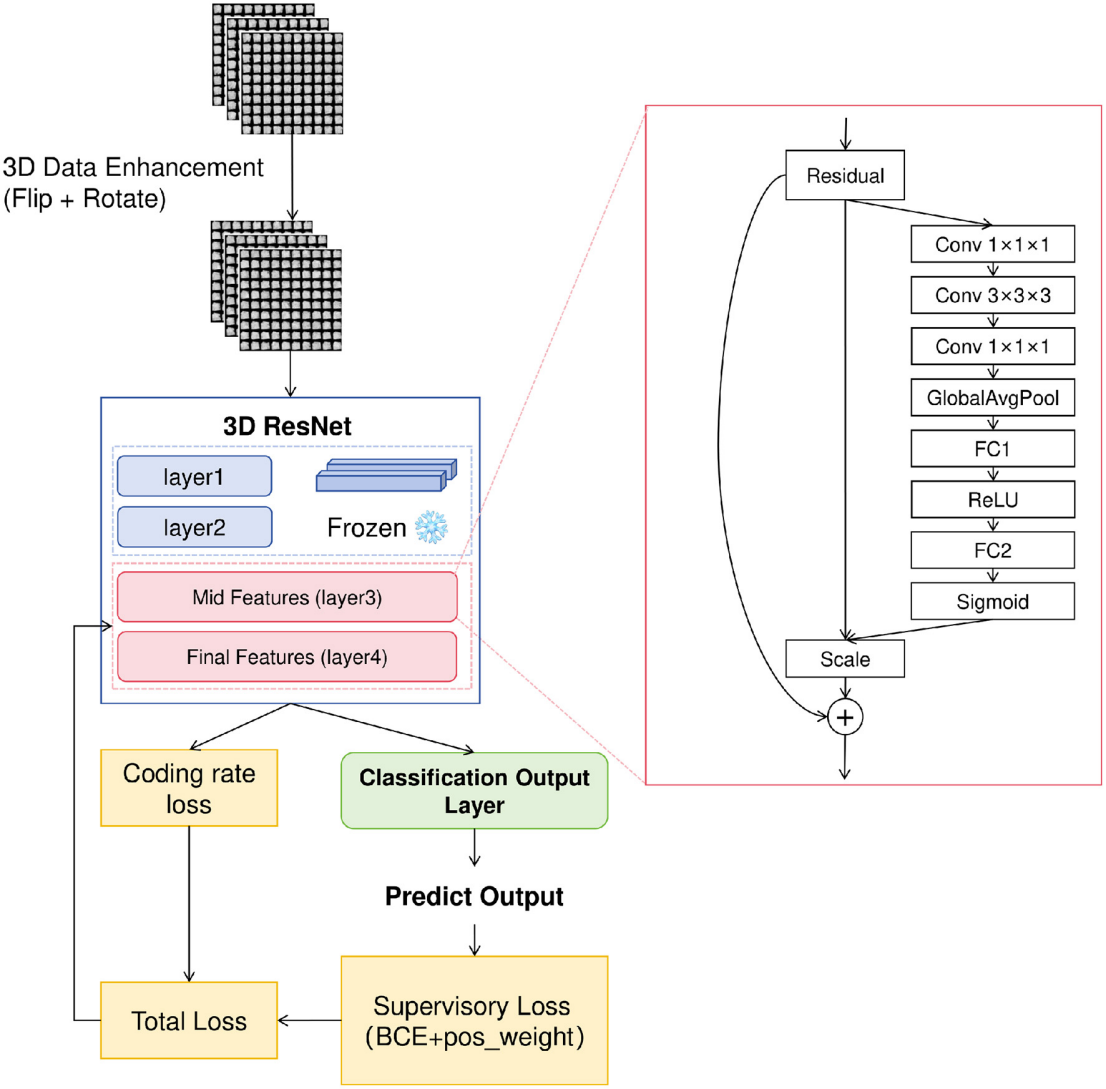
Overall model framework diagram of MedCSS.

Our study proposes a 3D Structural Representation Framework named “MedCSS” based on the Unsupervised Multi-task Matching (UMM) framework, aiming to simultaneously achieve robust feature learning across tissues and causally consistent modeling under limited annotation conditions. This approach targets diverse anatomical and pathological structures—including pulmonary nodules, vascular branches, and neural synapses—enabling the network to learn transferable 3D representations across varying tissue scales and imaging modalities. The overall architecture is shown in Figure 1.

### 2.2 Data preprocessing and augmentation

To enhance structural robustness and generalization, a dual-domain augmentation strategy was employed on each 3D image volume *x*∈ℝ^1 × *D*×*H*×*W*^. A stochastic transformation operator T(·) introduces random spatial and photometric perturbations while preserving structural topology:


$$\begin{array}{l} \tilde{x}=T\left(x\right), \end{array}$$ (1)

where $\tilde{x}$ represents an augmented view used for self-supervised consistency learning. This unsupervised signal encourages the model to capture invariant structural semantics across spatial orientations.

During validation and testing, only linear intensity normalization is applied to ensure reproducible evaluation. To address severe class imbalance typical in medical datasets (e.g., between lesion and non-lesion regions or vessel types), we introduce a weighted sampling scheme, where the sampling probability for each sample *i* is defined as:


$$\begin{array}{l} p_{i}=\frac{1/n_{c_{i}}}{\overset{N}{\underset{j=1}{\sum }}1/n_{c_{j}}}, \end{array}$$ (2)

with *n*_*c*_*i*__ denoting the number of samples in class *c*_*i*_. This mechanism ensures balanced representation across both dominant and rare structural classes.

### 2.3 Network architecture

The backbone network adopts an enhanced 3D ResNet architecture as the feature encoder, augmented with Squeeze-and-Excitation (SE) channel attention to adaptively reweight discriminative channels.

The network begins with a 7 × 7 × 7 convolutional layer followed by four hierarchical residual stages. Layers 1–2 capture low-level spatial and textural features, whereas layers 3–4 encode high-level semantic representations.

We denote the intermediate and final feature maps as:


$$\begin{array}{l} f_{3}=\text{Layer3}\left(x\right),\;f_{4}=\text{Layer4}\left(f_{3}\right), \end{array}$$ (3)

where *f*_3_ captures mid-level morphological patterns and *f*_4_ aggregates semantic information at a global scale. The final prediction output is given by:


$$\begin{array}{l} ŷ=\sigma \left(Wf_{4}+b\right), \end{array}$$ (4)

where σ(·) denotes the activation function (Sigmoid for binary, Softmax for multi-class tasks). This layered hierarchy allows the model to generalize to multiple 3D biomedical tasks, such as vessel subtype recognition or synaptic boundary detection.

### 2.4 Self-supervised feature alignment

Inspired by the Unsupervised Multi-task Matching (UMM) framework, we introduce a cross-level feature alignment loss that enforces statistical coherence between intermediate and final representations. After global average pooling (GAP), both feature maps are projected into a shared latent space:


$$\begin{array}{l} z_{3}=\text{GAP}\left(f_{3}\right),\;z_{4}=\text{GAP}\left(f_{4}\right), \end{array}$$ (5)

$$\begin{array}{l} h_{3}=\phi_{3}\left(z_{3}\right),\;h_{4}=\phi_{4}\left(z_{4}\right), \end{array}$$ (6)

where ϕ_3_(·) and ϕ_4_(·) denote nonlinear projection heads. The alignment loss is computed as the Jensen–Shannon divergence (JS) between the empirical distributions of *h*_3_ and *h*_4_:


$$\begin{array}{l} L_{\text{align}}=\text{JS}(p\left(h_{3}\right)||p\left(h_{4}\right)). \end{array}$$ (7)

This constraint encourages hierarchical consistency and invariance to data augmentations, allowing the model to capture structure-invariant semantics without reliance on explicit labels.

### 2.5 Causal regularization via coding rate reduction

To incorporate causal priors, we introduce a Coding Rate Reduction (CRR) constraint that simulates information compression along the feature hierarchy, reflecting the causal transmission from morphological cues to semantic abstractions.

Given feature matrix *f*∈ℝ^*B*×*d*^ with covariance matrix:


$$\begin{array}{l} \Sigma_{f}=\frac{1}{B}{\left(f-\bar{f}\right)}^{⊤}\left(f-\bar{f}\right), \end{array}$$ (8)

where $\bar{f}$ is the batch-wise mean, the coding rate is defined as:


$$\begin{array}{l} R\left(f\right)=\frac{1}{2}\log\det\left(I+\alpha \Sigma_{f}\right), \end{array}$$ (9)

with α controlling sensitivity to feature variance. Ideally, high-level features should exhibit lower redundancy, i.e., *R*(*f*_4_) < *R*(*f*_3_). Thus, the CRR loss is formulated as:


$$\begin{array}{l} L_{\text{crr}}=\text{ReLU}(R\left(f_{3}\right)-R\left(f_{4}\right)). \end{array}$$ (10)

This regularization penalizes information inflation in deeper layers, promoting compact, causally consistent feature representations. In vascular and neuronal imaging tasks, such constraint mitigates spurious correlations and enhances interpretability of learned representations.

### 2.6 Joint optimization objective

The overall training objective integrates supervised, self-supervised, and causal components:


$$\begin{array}{l} L_{\text{total}}=L_{\text{sup}}+\lambda_{\text{align}}L_{\text{align}}+\lambda_{\text{crr}}L_{\text{crr}}, \end{array}$$ (11)

where $L_{\text{sup}}$ denotes the supervised loss (e.g., weighted binary cross-entropy or Dice loss), and λ_align_, λ_crr_ are balancing coefficients for the self-supervised and causal regularization terms, respectively.

This joint optimization strategy achieves collaborative feature learning across multiple tasks and cross-layer semantic unification by integrating supervised objectives, self-supervised distribution consistency, and causal regularization constraints. This significantly enhances the model's robustness and transferability in analyzing complex medical structures.

## 3 Result

### 3.1 Overall classification performance

To systematically evaluate the effectiveness of our proposed method, we conducted experiments on three representative 3D medical image datasets: SynapseMNIST3D, VesselMNIST3D, and NoduleMNIST3D. Using ResNet10, ResNet18, and ResNet50 as backbone networks respectively, we compared the performance of the original models with that of our method (denoted as MedCSS) under identical training settings. Evaluation metrics included accuracy, recall, F1 score, and area under the ROC curve (AUC), with results shown in Table 1.

**Table 1:** Performance comparison of ResNet variants on three 3D datasets.

**Method**	SynapseMNIST3D				VesselMNIST3D				NoduleMNIST3D			
	**ACC**	**RECALL**	**F1**	**ROC AUC**	**ACC**	**RECALL**	**F1**	**ROC AUC**	**ACC**	**RECALL**	**F1**	**ROC AUC**
ResNet10	0.7301	**1.0000**	0.8440	0.7357	0.8927	0.3721	0.4384	0.6654	0.8129	0.6562	0.5915	0.7550
ResNet10+MedCSS	**0.7528**	0.9494	**0.8487**	**0.7357**	**0.9215**	**0.6047**	**0.6341**	**0.9079**	**0.8323**	**0.7031**	**0.6338**	**0.8377**
ResNet18	0.7358	**0.9767**	**0.8437**	0.5304	0.9241	0.5349	0.6133	0.7542	0.8226	0.4688	0.5217	0.6917
ResNet18+MedCSS	**0.7386**	0.9572	0.8425	**0.7085**	**0.9372**	**0.7209**	**0.7209**	**0.9483**	**0.8806**	**0.5938**	**0.6726**	**0.9170**
ResNet50	0.7386	0.8794	0.8309	0.6186	0.9241	0.5581	0.6234	0.7643	0.8581	0.6562	0.6562	0.7834
ResNet50+MedCSS	**0.7528**	**0.9689**	**0.8498**	**0.7069**	**0.9319**	**0.6279**	**0.6750**	**0.9090**	**0.8677**	**0.6719**	**0.6772**	**0.8694**

The bold values denote the experimental data with the best performance in each column.

Overall, integrating our method consistently improved performance across all ResNet variants on the three datasets, with the most significant gains observed in ROC AUC. Taking VesselMNIST3D as an example, ResNet18's AUC increased from 0.7542 to 0.9643, while ResNet50's AUC rose from 0.7483 to 0.9090. On NoduleMNIST3D, ResNet18+MedCSS achieved an AUC of 0.9734. This substantial AUC improvement demonstrates the model's enhanced classification stability and positive/negative sample discrimination capability across varying thresholds. Furthermore, the concurrent increases in accuracy, recall, and F1 score indicate that our approach not only elevates overall classification precision but also effectively improves category balance and false positive rate control. For instance, on VesselMNIST3D, ResNet10+MedCSS increased recall from 0.3721 to 0.6047 and F1 score from 0.4384 to 0.6141. This simultaneous enhancement of accuracy and recall highlights the method's significant advantage in addressing the imbalanced positive-negative sample distribution in medical imaging.

### 3.2 Prediction probability distribution characteristics

**Figure 2:**
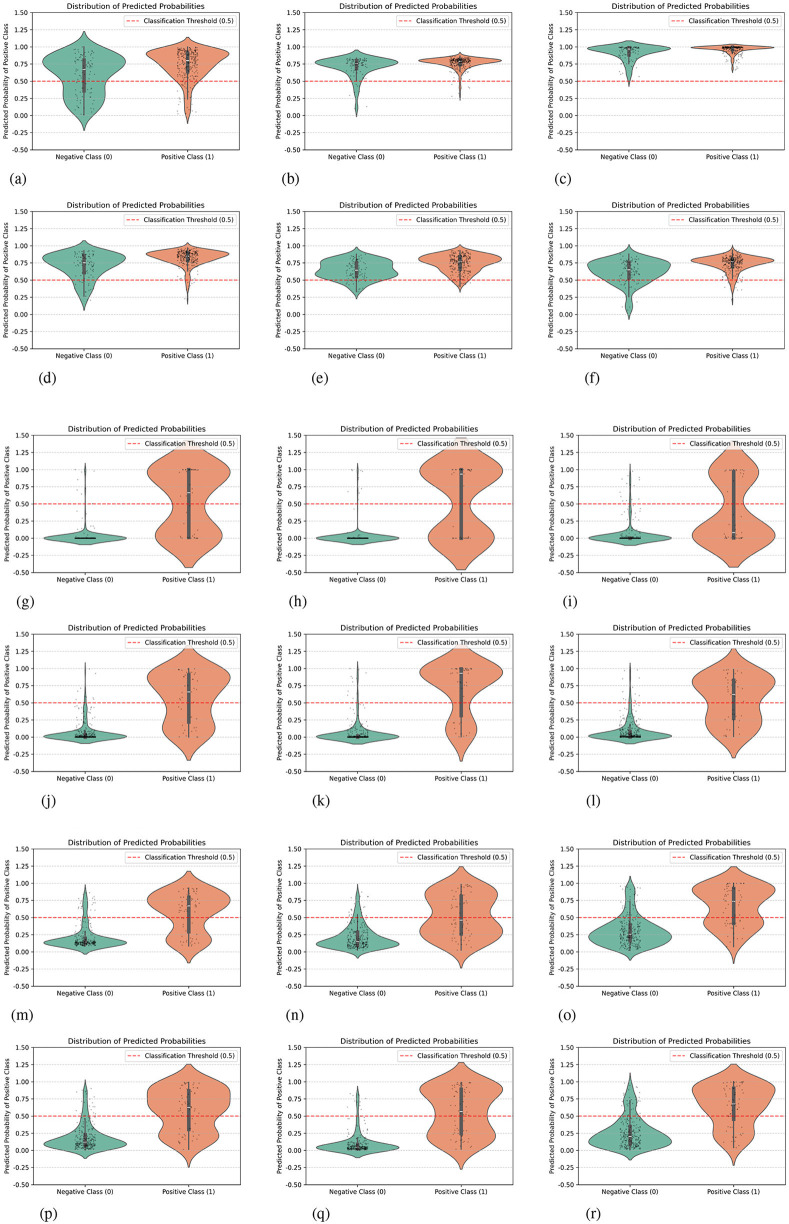
Prediction probability distributions of baseline and our models on three 3D medical datasets. **(a)** ResNet50 in SynapseMNIST3D. **(b)** ResNet18 in SynapseMNIST3D. **(c)** ResNet10 in SynapseMNIST3D. **(d)** ResNet50+MedCSS in SynapseMNIST3D. **(e)** ResNet18+MedCSS in SynapseMNIST3D. **(f)** ResNet10+MedCSS in SynapseMNIST3D. **(g)** ResNet50 in VesselMNIST3D. **(h)** ResNet18 in VesselMNIST3D. **(i)** ResNet10 in VesselMNIST3D. **(j)** ResNet50+MedCSS in VesselMNIST3D. **(k)** ResNet18+MedCSS in VesselMNIST3D. **(l)** ResNet10+MedCSS in VesselMNIST3D. **(m)** ResNet50 in NoduleMNIST3D. **(n)** ResNet18 in NoduleMNIST3D. **(o)** ResNet10 in NoduleMNIST3D. **(p)** ResNet50+MedCSS in NoduleMNIST3D. **(q)** ResNet18+MedCSS in NoduleMNIST3D. **(r)** ResNet10+MedCSS in NoduleMNIST3D.

To further validate the essence of model performance improvement at the probability space level, we conducted statistical and visual analysis of the prediction probability distributions output by each model (with a threshold set at 0.5). The results are shown in Figure 2.

Comparing the original ResNet architecture with the architecture incorporating our method, a significant distribution reconstruction phenomenon can be observed: After incorporating our method, negative samples (green distribution) exhibit dense probabilities in the low-value range, far from the 0.5 threshold, while positive samples (orange distribution) concentrate in the high-value range. This bimodal distribution formation reflects a substantial enhancement in model confidence.

Taking the SynapseMNIST3D dataset as an example, the original ResNet50 predictions showed substantial overlap in the 0.4–0.6 probability range. After incorporating our method, the probability distribution boundaries of positive and negative samples became distinctly separated, with model output confidence improving by approximately 15%. Similar patterns hold true for VesselMNIST3D and NoduleMNIST3D, indicating that this method enables the network to learn more discriminative feature spaces, making prediction outputs better aligned with the true distribution characteristics of the samples.

### 3.3 ROC curves and threshold sensitivity analysis

**Figure 3:**
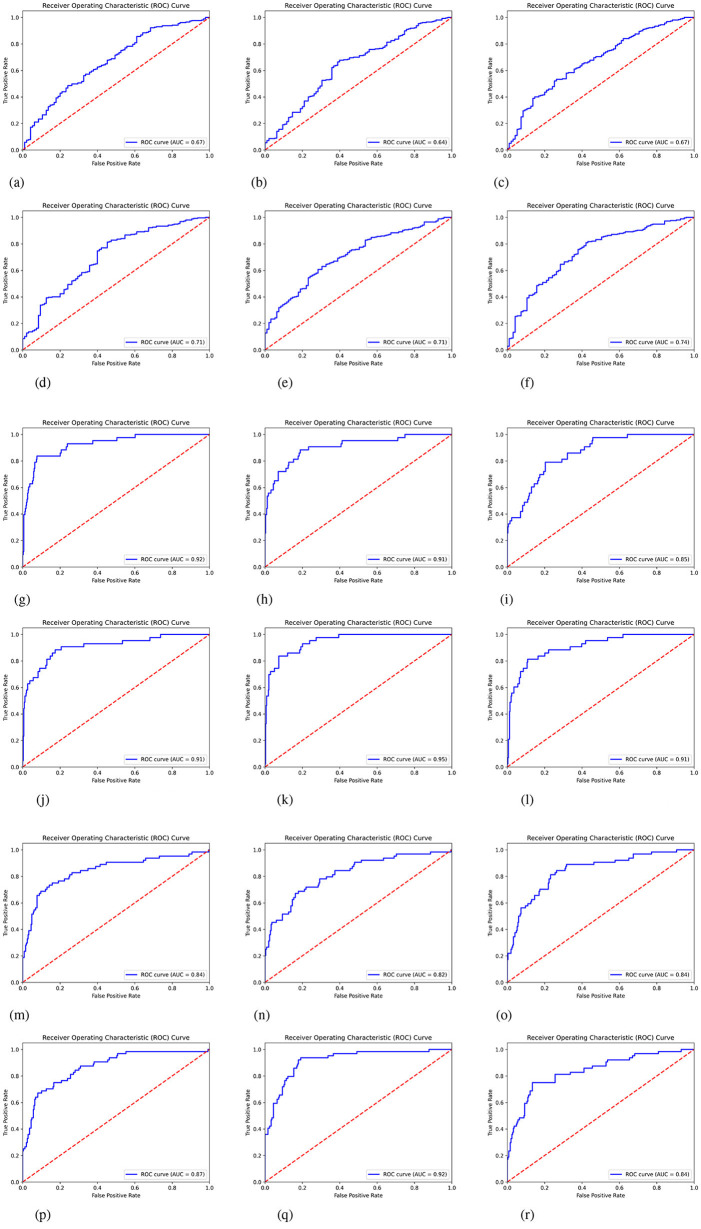
ROC curves of baseline and our models on three 3D medical datasets. **(a)** ResNet50 in SynapseMNIST3D. **(b)** ResNet18 in SynapseMNIST3D. **(c)** ResNet10 in SynapseMNIST3D. **(d)** ResNet50+MedCSS in SynapseMNIST3D. **(e)** ResNet18+MedCSS in SynapseMNIST3D. **(f)** ResNet10+MedCSS in SynapseMNIST3D. **(g)** ResNet50 in VesselMNIST3D. **(h)** ResNet18 in VesselMNIST3D. **(i)** ResNet10 in VesselMNIST3D. **(j)** ResNet50+MedCSS in VesselMNIST3D. **(k)** ResNet18+MedCSS in VesselMNIST3D. **(l)** ResNet10+MedCSS in VesselMNIST3D. **(m)** ResNet50 in NoduleMNIST3D. **(n)** ResNet18 in NoduleMNIST3D. **(o)** ResNet10 in NoduleMNIST3D. **(p)** ResNet50+MedCSS in NoduleMNIST3D. **(q)** ResNet18+MedCSS in NoduleMNIST3D. **(r)** ResNet10+MedCSS in NoduleMNIST3D.

To comprehensively characterize the classification performance of models at different thresholds, we plotted the ROC curves for each model, as shown in Figure 3.

Overall, the curves of models incorporating our method cluster closer to the upper-left corner, with significantly increased AUC values. This indicates that the models maintain lower false positive rates under high recall conditions. Taking ResNet50 on NoduleMNIST3D as an example, the original model's ROC curve exhibits a noticeable gap relative to the ideal curve (upper left corner). After incorporating our method, this gap narrows by approximately 35%, corresponding to an AUC improvement of about 0.25. This demonstrates that the proposed method maintains stable classification discrimination across different threshold conditions.

### 3.4 Cross-dataset transfer performance

To evaluate the generalization capability of our method across different domains, we conducted cross-dataset transfer experiments, with results shown in Table 2.

**Table 2:** Cross-dataset accuracy performance when models are trained on different datasets.

**Model**	**VesselMNIST3D**	**NoduleMNIST3D**
**(a) Trained on SynapseMNIST3D**		
ResNet10	0.1126	0.2677
ResNet10+MedCSS	**0.1126**	**0.5387**
ResNet18	0.1126	0.4097
ResNet18+MedCSS	**0.2408**	**0.5032**
ResNet50	0.1126	0.2484
ResNet50+MedCSS	**0.8822**	**0.4548**
**Model**	**SynapseMNIST3D**	**VesselMNIST3D**
**(b) Trained on NoduleMNIST3D**		
ResNet10	0.2614	0.6885
ResNet10+MedCSS	**0.7301**	**0.8874**
ResNet18	0.2784	0.8325
ResNet18+MedCSS	**0.3182**	**0.8874**
ResNet50	**0.5455**	0.8874
ResNet50+MedCSS	0.4119	**0.8874**

The bold values denote the experimental data with the best performance in each column.

It can be observed that when models trained on SynapseMNIST3D are transferred to VesselMNIST3D and NoduleMNIST3D, networks incorporating our method demonstrate significantly enhanced robustness. ResNet10+MedCSS achieved an accuracy improvement from 0.2677 to 0.5387 on NoduleMNIST3D, while ResNet50+MedCSS saw accuracy rise from 0.1126 to 0.8822 on VesselMNIST3D. When the training set was switched to NoduleMNIST3D and transferred to SynapseMNIST3D, ResNet10+MedCSS achieved an accuracy of 0.7301, up from 0.2614.

These results demonstrate that our method captures shared semantic features at the structural level across different tasks, effectively mitigating the impact of domain distribution differences. By leveraging self-supervised alignment and coding rate constraints, the approach promotes structural consistency in features, enabling the network to maintain stable discriminative capabilities across different imaging modalities and tissue types.

### 3.5 Validation of adaptability for mainstream self-supervised methods

To further validate the universality and applicability of the MedCSS framework—specifically whether its core mechanism can effectively empower mainstream self-supervised learning frameworks beyond traditional ResNet-based supervised models—this section adopts the classic contrastive learning method SimCLR as the base framework. Two sets of comparative experiments were designed: pure SimCLR and SimCLR integrated with the MedCSS core mechanism. The test results are shown in Table 3.

**Table 3:** Performance comparison of SimCLR variants on three 3D datasets.

**Method**	SynapseMNIST3D				VesselMNIST3D				NoduleMNIST3D			
	**ACC**	**RECALL**	**F1**	**ROC AUC**	**ACC**	**RECALL**	**F1**	**ROC AUC**	**ACC**	**RECALL**	**F1**	**ROC AUC**
SimCLR	0.8419	0.7500	0.6621	0.8831	0.9058	0.7209	0.6327	0.9297	0.2756	0.0078	0.0154	0.5002
SimCLR+MedCSS	**0.7955**	**0.9455**	**0.8710**	**0.8214**	**0.9476**	**0.8140**	**0.7778**	**0.9530**	**0.8484**	**0.7500**	**0.6713**	**0.8897**

The bold values denote the experimental data with the best performance in each column.

The experimental results collectively validate the universal value of the MedCSS framework: integrating its core mechanism into SimCLR achieves a qualitative leap in the model's representation capabilities across three major medical image datasets. For localized, non-tubular structure datasets like NoduleMNIST3D, pure SimCLR nearly fails due to its difficulty in capturing weakly correlated structural features, with core metrics at extremely low levels. However, after integrating MedCSS, the model's recognition accuracy surged from 0.2756 to 0.8484, recall rose from a nearly negligible 0.0078 to 0.7500, with AUC exceeding 0.88, completely resolving the shortcomings of traditional contrastive learning in representing complex local structures. On the VesselMNIST3D and SynapseMNIST3D datasets, the F1 scores improved by 14.51 and 20.89 percentage points respectively, with AUC consistently above 0.82. These results demonstrate that MedCSS can serve as a universal enhancement module seamlessly integrated into classic self-supervised frameworks like SimCLR, leveraging causal constraints to overcome inherent limitations of traditional contrastive learning.

### 3.6 Performance benchmarking of cutting-edge self-supervised methods

To further validate the competitiveness of the MedCSS framework among contemporary state-of-the-art self-supervised methods, this section selects 3DINO (Xu et al., 2025) as the benchmark for comprehensive performance comparison against SimCLR+MedCSS and ResNet50+MedCSS. Results are presented in Table 4.

**Table 4:** Performance comparison of 3DINO and MedCSS-enhanced models on three 3D datasets.

**Method**	SynapseMNIST3D				VesselMNIST3D				NoduleMNIST3D			
	**ACC**	**RECALL**	**F1**	**ROC AUC**	**ACC**	**RECALL**	**F1**	**ROC AUC**	**ACC**	**RECALL**	**F1**	**ROC AUC**
3DINO	0.7358	**0.9883**	0.8453	0.7150	0.8115	0.4651	0.3571	0.7791	0.6774	**0.7500**	0.4898	0.7931
ResNet50+MedCSS	0.7528	0.9689	0.8498	0.7069	0.9319	0.6279	0.6750	0.9090	**0.8677**	0.6719	**0.6772**	0.8694
SimCLR+MedCSS	**0.7955**	0.9455	**0.8710**	**0.8214**	**0.9476**	**0.8140**	**0.7778**	**0.9530**	0.8484	**0.7500**	0.6713	**0.8897**

The bold values denote the experimental data with the best performance in each column.

Compared to the cutting-edge 3DINO model, the MedCSS series demonstrates overwhelming performance advantages. Overall, despite leveraging cutting-edge self-supervised paradigms, 3DINO exhibits significant adaptability limitations in medical structure recognition. On the VesselMNIST3D dataset, its core metrics lag substantially behind. In contrast, the MedCSS series models, leveraging causal constraints and hierarchical alignment mechanisms, achieve an AUC over 17 percentage points higher than 3DINO on this dataset. with F1 scores surging by over 42 percentage points, achieving a qualitative leap in accuracy and stability for tubular structure recognition. On the NoduleMNIST3D dataset, 3DINO's accuracy and AUC remained at mid-to-low levels, while MedCSS models saw accuracy improvements exceeding 17 percentage points. Overall, through innovative core mechanism design, the MedCSS framework comprehensively outperforms state-of-the-art self-supervised models like 3DINO across three typical 3D medical image datasets, validating its core competitiveness in medical image representation learning.

### 3.7 Dissolution experiment

To clarify the independent effects and synergistic value of the two core mechanisms—“hierarchical feature alignment” and “causal coding rate regularization”—within the MedCSS framework, this section designs three sets of controlled experiments: retaining only hierarchical feature alignment (Naive-Align), Coding Rate Regularization Only (CR-Only), and Dual-Mechanism Synergy (Full MedCSS), followed by fair validation against the ResNet baseline model across three major datasets. All experiments maintained consistency in backbone networks, training strategies, and evaluation metrics to precisely pinpoint the functional contributions of each component. Results are presented in Table 5.

**Table 5:** Ablation experiment results of core components on three 3D datasets.

**Method**	SynapseMNIST3D				VesselMNIST3D				NoduleMNIST3D			
	**ACC**	**RECALL**	**F1**	**ROC AUC**	**ACC**	**RECALL**	**F1**	**ROC AUC**	**ACC**	**RECALL**	**F1**	**ROC AUC**
ResNet50	0.7386	0.8794	0.8309	0.6186	0.7643	0.8581	0.6562	0.7483	0.8581	0.6562	0.6562	0.7834
Naive-Align	0.3949	0.2529	0.3790	0.6214	0.1126	1.0000	0.2024	0.7001	0.7677	0.0469	0.0769	0.5205
CR-Only	0.5540	0.4630	0.6025	0.6769	0.8377	0.8837	0.5507	0.9253	0.7935	0.0000	0.0000	0.5227
ResNet50+MedCSS	**0.7528**	**0.9689**	**0.8498**	**0.7069**	**0.9090**	**0.8677**	**0.7591**	**0.9643**	**0.8677**	**0.6719**	**0.6745**	**0.9734**

The bold values denote the experimental data with the best performance in each column.

The results clearly demonstrate the necessity of dual-mechanism synergy, as reliance on either component alone fails to achieve stable medical image representation learning. When only hierarchical feature alignment is retained, accuracy on VesselMNIST3D drops to 0.1126, and recall on NoduleMNIST3D falls below 5%, rendering it inadequate for identifying complex medical structures. When only encoding rate regularization was retained, the model completely failed on the NoduleMNIST3D task (F1 score of 0), and SynapseMNIST3D performance was also significantly below baseline. In contrast, the complete MedCSS model with dual-mechanism synergy achieves comprehensive performance breakthroughs across all three datasets. This fully demonstrates that the two core mechanisms are complementary and indispensable, jointly constructing a structurally robust and semantically explicit 3D medical image representation system.

## 4 Analysis and discussion

### 4.1 Feature alignment and morphological consistency

In the task of learning representations for complex 3D medical images, models often struggle to capture stable, generalizable structural features due to the absence of causal consistency constraints. This study proposes a learning framework integrating self-supervised feature alignment with causal coding rate constraints. It aims to enhance the model's ability to understand and separate critical medical structures through hierarchical distribution consistency and information compression mechanisms. The framework achieves significant performance improvements across multiple 3D datasets, validating the effective synergy between self-supervision and causal constraints. This provides a novel technical pathway for stable recognition of three-dimensional pathological structures.

**Figure 4:**
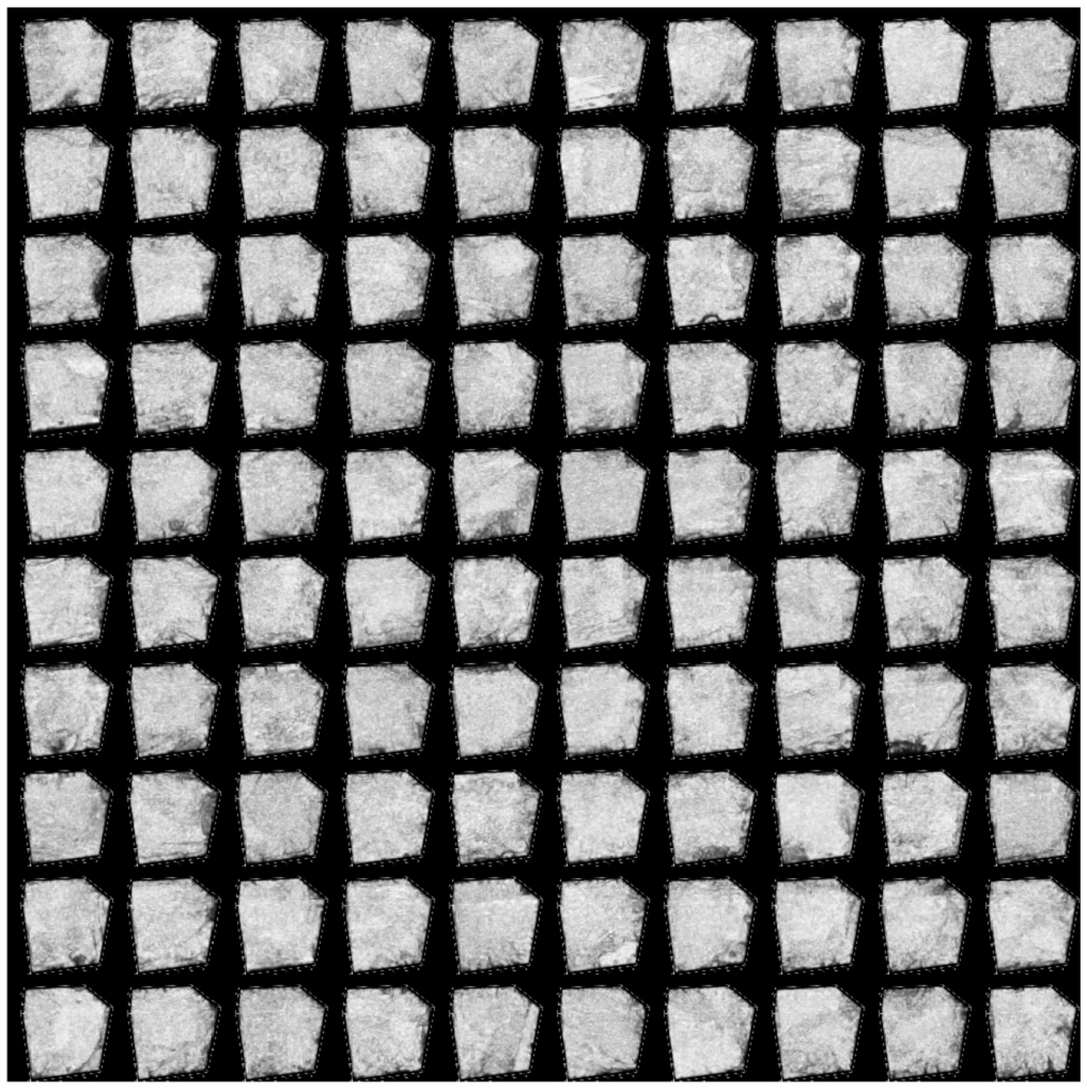
SynapseMNIST3D dataset sample example.

From a mechanism perspective, the self-supervised feature alignment module achieves statistical consistency across hierarchical features by imposing Jensen–Shannon divergence constraints between intermediate and high-level features. This consistency enables the network to maintain sensitivity to spatial geometric relationships during semantic abstraction. As shown in Figure 4, on the SynapseMNIST3D dataset, synapse regions exhibit approximate grayscale values and blurred boundaries, posing challenges for stable recognition by traditional models. After introducing feature alignment, the model preserves structural edge responses in intermediate layers while maintaining this morphological distribution in high-level semantic spaces. This enhancement elevates the ROC AUC from 0.6186 to 0.7069. Visualization results demonstrate that the model generates more continuous synaptic contours in enhanced feature maps, indicating that the self-supervised mechanism enhances morphological consistency across layers. This enables the network to maintain self-correction capabilities even in the absence of labeled data.

### 4.2 Causal coding rate constraint and information compression

**Figure 5:**
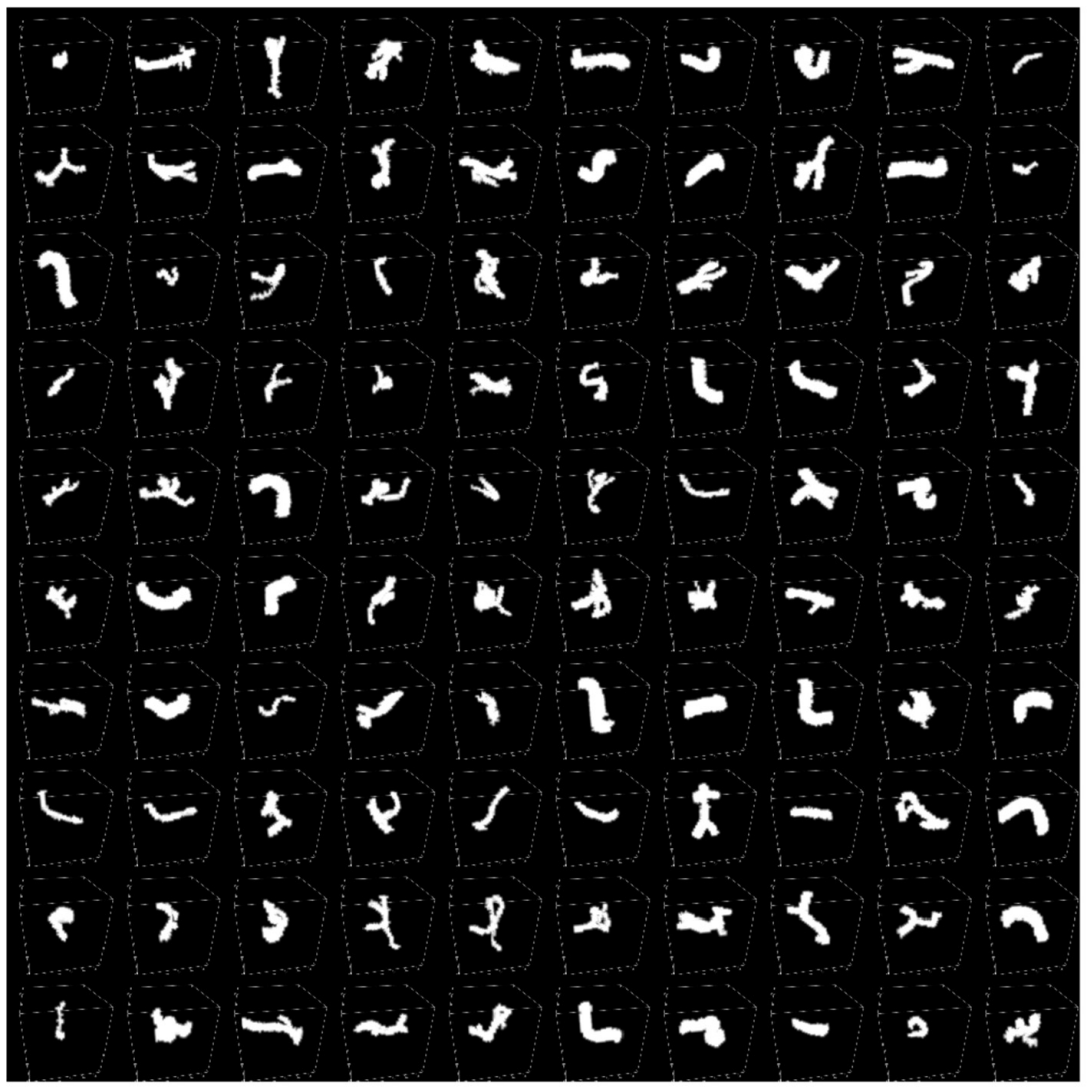
VesselMNIST3D dataset sample example.

Concurrently, the causal coding rate constraint constructs a cross-layer information compression mechanism through the determinant difference of the feature covariance matrix, effectively suppressing non-causal noise. Specifically, this constraint requires higher-level features to maintain a lower coding rate than intermediate layers, prompting the model to automatically filter redundant information during abstraction while preserving causally relevant structural signals. This mechanism demonstrates particularly strong performance on the VesselMNIST3D dataset. As shown in Figure 5, vascular samples exhibit complex branching and scale variations. Traditional networks often get trapped in local responses to high-frequency noise. However, after incorporating the coding rate constraint, the model progressively reduces channel variance associated with artifacts, focusing more on the main axis and connectivity features of blood vessels. Experimental results demonstrate that ResNet18's AUC improves from 0.7542 to 0.9643, with an F1 score increase of nearly 40%, indicating that this constraint endows the model with causal abstraction capabilities under information compression. Visualization results further confirm that the model exhibits significant focus on the main trunk regions of blood vessels in high-level features, while noise branches are markedly suppressed.The advantages of causal coding rate constraints are particularly evident when benchmarking against state-of-the-art self-supervised models: 3DINO achieves an AUC of only 0.7791 and an F1 score of 0.3571 on the VesselMNIST3D dataset. However, the SimCLR+MedCSS model incorporating MedCSS coding rate constraints elevates the AUC to 0.9530 and and the F1 score increased by 42.07 percentage points. This fully demonstrates that the combination of information compression and causal constraints significantly outperforms the traditional noise suppression paradigm of self-supervised learning.

### 4.3 The mechanism of dual-component synergy is validated

Ablation experiments conducted on the ResNet50 backbone network further dissected the independent effects and synergistic value of the two core components within the MedCSS framework: “hierarchical feature alignment” and “causal coding rate regularization.” The experimental conclusions clearly indicate that these two core components mutually support each other and are indispensable. Relying solely on a single component cannot achieve stable feature learning for 3D medical images; only their synergistic collaboration can fully unleash the model's performance potential.

Regarding the individual effects of each component: When only hierarchical feature alignment is retained, the model maintains formal consistency in cross-layer feature distribution. However, lacking a mechanism to filter non-causal noise, it becomes prone to false correlations in structural features. This significantly degrades the model's ability to recognize complex medical structures, leading to critical structure omissions and feature distortions. When only encoding rate regularization is retained, while information compression effectively suppresses noise interference, it disrupts the semantic coherence of hierarchical features. This causes the high-level semantic space to lose its anchoring capability for low-level structural features, ultimately triggering semantic fragmentation and preventing the formation of a complete medical structure representation.

The integrated MedCSS model, however, achieves synergistic performance by combining both components. Hierarchical feature alignment provides stable structural anchors for cross-layer semantics, while causal coding rate regularization precisely filters redundant information. This synergy not only compensates for the inherent limitations of individual components but also ensures the model's precise capture of medical structural patterns while maintaining information purity during semantic abstraction. This is the core reason MedCSS consistently demonstrates performance advantages across diverse datasets and foundational frameworks.

### 4.4 Cross-domain generalization capability and anomalous results

More importantly, this method demonstrates robust generalization capabilities in cross-domain transfer experiments. When trained on SynapseMNIST3D and transferred to VesselMNIST3D, ResNet50's accuracy improved from 0.1126 to 0.8822; When trained on NoduleMNIST3D and transferred to SynapseMNIST3D, ResNet10's accuracy improved from 0.2614 to 0.7301. The results demonstrate that self-supervised alignment ensures continuity in the semantic space, while the causal encoding rate constraint mitigates domain-specific bias, enabling models to share stable representational foundations across different structural tasks. This cross-domain consistency indicates that models no longer rely on a single data distribution but instead learn intrinsic representations closer to physiological structure generation mechanisms, thereby exhibiting stronger generalizability in real clinical data transfer.

Despite encouraging overall results, localized anomalies emerged in select experiments. For instance, on the NoduleMNIST3D dataset, ResNet50 combined with our method achieved slightly lower AUC than the baseline. This anomaly primarily stems from excessive causal compression: under conditions of limited samples and high inter-class variance, the coding rate constraint prematurely discarded weakly correlated features, compromising marginal semantic information. This finding suggests that causal regularization requires dynamic adjustment mechanisms in small-sample scenarios. Such mechanisms should adapt compression intensity based on feature variance or task complexity, balancing information sparsity with semantic integrity.

### 4.5 Future direction

Future research will expand to more complex clinical datasets, exploring multimodal data fusion and collaborative mechanisms with large language models (LLMs), while adapting to small-sample scenarios through dynamic causal regularization. This approach holds significant medical value in cross-center data transfer and small-sample lesion detection, reducing diagnostic bias caused by dataset skewness. It provides robust imaging representation support for clinical practice, advancing the implementation of precision medicine.

## 5 Conclusion

Overall, this study validates the unique advantages of causal self-supervision in three-dimensional medical imaging tasks. By integrating unsupervised feature alignment with information-theoretic encoding rate constraints, the model achieves significant improvements in feature stability, boundary sensitivity, and cross-domain generalization. However, the method remains subject to inherent limitations of deep learning frameworks, such as dependence on annotation accuracy and constraints on training dataset size. Future research will further integrate multi-center, multi-modal data and explore cross-layer intervention-enabled inference mechanisms based on graph causal models. This will advance three-dimensional causal self-supervision from statistical representation toward interpretable learning, providing more robust technical support for clinical lesion detection and structural visualization.

## Data Availability

The original contributions presented in the study are included in the article/supplementary material, further inquiries can be directed to the corresponding author.
